# The metaphyseal sleeve: an unexplored option in the treatment of complex primary knee osteoarthritis

**DOI:** 10.1186/s43019-020-00032-9

**Published:** 2020-04-16

**Authors:** W. K. Wong, H. S. Chua

**Affiliations:** 1 National Orthopaedic Centre of Excellence for Research and Learning (NOCERAL), Univeristy of Malaya, Kuala Lumpur, Malaysia; 2 Department of Orthopaedics, Hospital Pulau Pinang, Penang, Malaysia

**Keywords:** Knee, Complex primary osteoarthritis, Metaphyseal sleeve

## Abstract

**Background:**

In an ever-aging society that as a whole has become more affluent, significant emphasis has been accorded to an improved quality of life. Knee osteoarthritis is ever-increasingly treated with total knee arthroplasty. The benefits and satisfaction experienced by those who have undergone total knee replacements (TKR) are well documented in the literature. The issue arises when osteoarthritis of the affected knee is more complex than simple osteoarthritis, i.e. the patient has complex primary osteoarthritis. This collective term encompasses conditions such as massive bone loss, ligamentous laxity, coronal defects and those with contractures. There are various classifications to describe massive bone loss but we utilized the Anderson Orthopaedic Research Institute (AORI) classification. Numerous treatment options are available and we report the use of metaphyseal sleeves as a highly successful treatment option.

**Methods:**

We retrospectively reviewed all the patients at our centre who underwent primary TKR using the metaphyseal sleeves. Patients were assessed on symptoms and functional status, and radiographs were also taken to assess for osseointegration. Only patients who completed 2 years of follow up were included in our study.

**Results:**

The updated (2011) Knee Society Score (KSS) was used in conjunction with radiological assessments at each follow up. Mean KSS scores improved from 53.83 preoperatively to 193.39 postoperatively. All patients demonstrated increasing osseointegration throughout follow up.

**Conclusion:**

The metaphyseal sleeve is an excellent treatment option for complex primary osteoarthritic knees with good results objectively, functionally and radiologically and would be a great choice for all orthopaedic surgeons to include in their armamentarium.

## Introduction

**Fig. 1:**
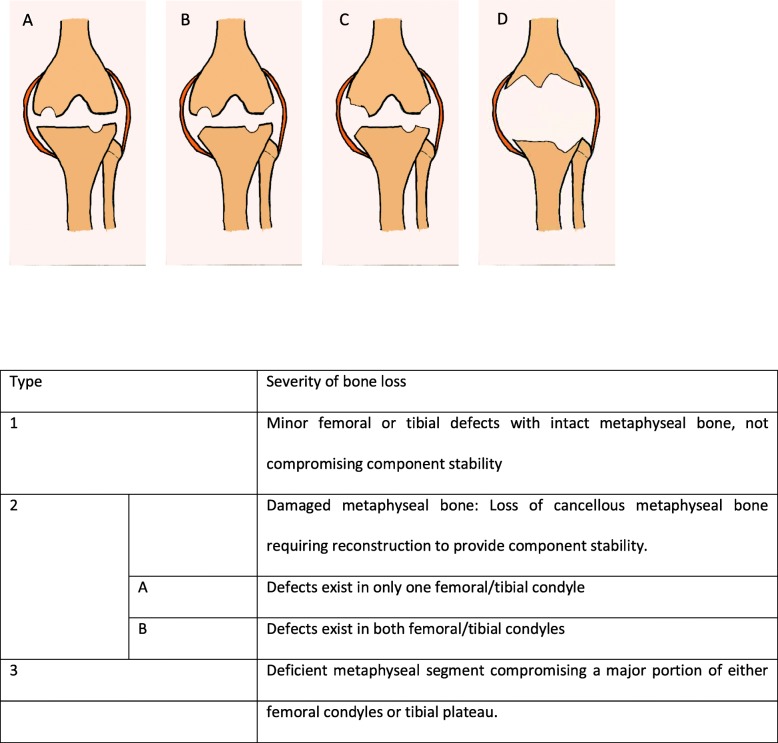
Anderson Orthopaedic Research Institute (AORI) classification of bone defects with description (in table). **a** type 1 defect; **b** type 2A defect; **c** type 2B defect; **d** type 3 defect

Health advancements have grown tremendously and this is mirrored by the increasing life expectancy of society as a whole (Riley [1]). Given this and the fact that the general population is more affluent and places ever-increasing emphasis on quality of life, it would then not be surprising to note that the frequency of total knee replacements (TKR) continues to increase. The projected number of TKR per year is expected to be upwards of 3 million in the USA alone by the year 2030 (Kurtz et al. [2]). The proven benefits of TKR in the treatment of osteoarthritis and its reproducibility makes the procedure an attractive treatment option (Meding et al. [3]). The issue arises when the patient has complex primary osteoarthritis of the knee (characterised by massive bone loss, ligamentous laxity, coronal defects or contractures (Baldini et al. [4])) rather than simple primary osteoarthritis. For the purpose of this paper, the issue being addressed would be the massive bone loss. Bone loss is commonly classified using the Anderson Orthopaedic Research Institute (AORI) Classification, which was intended for use in revision TKR, but we have extrapolated it for use in our series of complex primary TKR (Qiu et al. [5], Engh [6]). The AORI approach is used to classify femoral and tibial defects as type 1, 2 or 3. (Fig. 1). In type 1 defects, the metaphyseal bone is intact and component stability is preserved. In type 2 defects, the metaphyseal bone is damaged and there is loss of cancellous bone in either one femoral/tibial condyle (type 2A) or both condyles (type 2B). Type 3 defects are characterised by deficient metaphyseal bone and would traditionally require use of structural allografts, hinged prostheses and revision knee systems (Qiu et al. [5], Engh [6]). Treatment options in the setting of revision TKR include use of bone cement either on its own or in conjunction with screws, bone grafting, metal augments (long stems, wedge augments, tantalum cones, metaphyseal sleeves), structural allografts, hinged prostheses and custom-made implants. The decision on which treatment option is to be employed is made on a case-by-case basis and is highly dependent on the surgical expertise available. The metaphyseal sleeve has gained some traction in the revision setting but has not yet been reported as a treatment alternative in complex primary osteoarthritis. Our hypothesis is that the metaphyseal sleeve is a viable treatment option for complex primary knee osteoarthritis. The current series represent the only report, to the author’s knowledge, of the use of metaphyseal sleeves in the management of complex primary TKRs.

## Materials and methods

Institutional Review Board approval was obtained from the Medical Research & Ethics Committee of the Malaysian Ministry of Health (NMRR-18-339-39,604(IIR)). This study is a retrospective study utilising data collected from the office of the senior author (HS Chua) who personally conducted all assessments and examinations on the patients. Patients were followed up at our centre at 2 weeks, 6 weeks, 3 months and 6 months post-surgery and thereafter half-yearly. Patients were assessed on symptoms, functional status and radiographs (antero-posterior (AP) projection with the patient standing and lateral projection radiographs) were also taken to assess osseointegration and alignment. The degree of varus or valgus present is calculated based on the angulation subtended by the femoral component and tibial axis, which is taken as a line drawn through the midpoints along the tibial shaft.

Case files for all the patients were traced and reviewed. Parameters of interest included the patient’s gender, age, laterality (right, left or bilateral), AORI grade and preoperative and postoperative range of motion, degree of varus/valgus deformity and KSS scores (Scuderi et al. [7]). Our centre has used the new KSS scoring system for all our patients undergoing joint replacements as it is a validated scoring system that takes into account both objective (alignment, stability and range of motion) and patient-reported subjective outcomes (symptoms, expectations, satisfaction and activity levels). The objective elements of the KSS were completed by the senior author during clinic consults. Inclusion criteria are patients with severe knee osteoarthritis who have undergone primary TKR, i.e. not a revision procedure, and who received the metaphyseal sleeve implant (Sigma TC3 & MBT Revision Knee System, Depuy Synthes, Johnson & Johnson). Exclusion criteria were patients who underwent primary TKR using the metaphyseal sleeve, who have yet to complete 2 years of postoperative follow up.

**Fig. 2:**
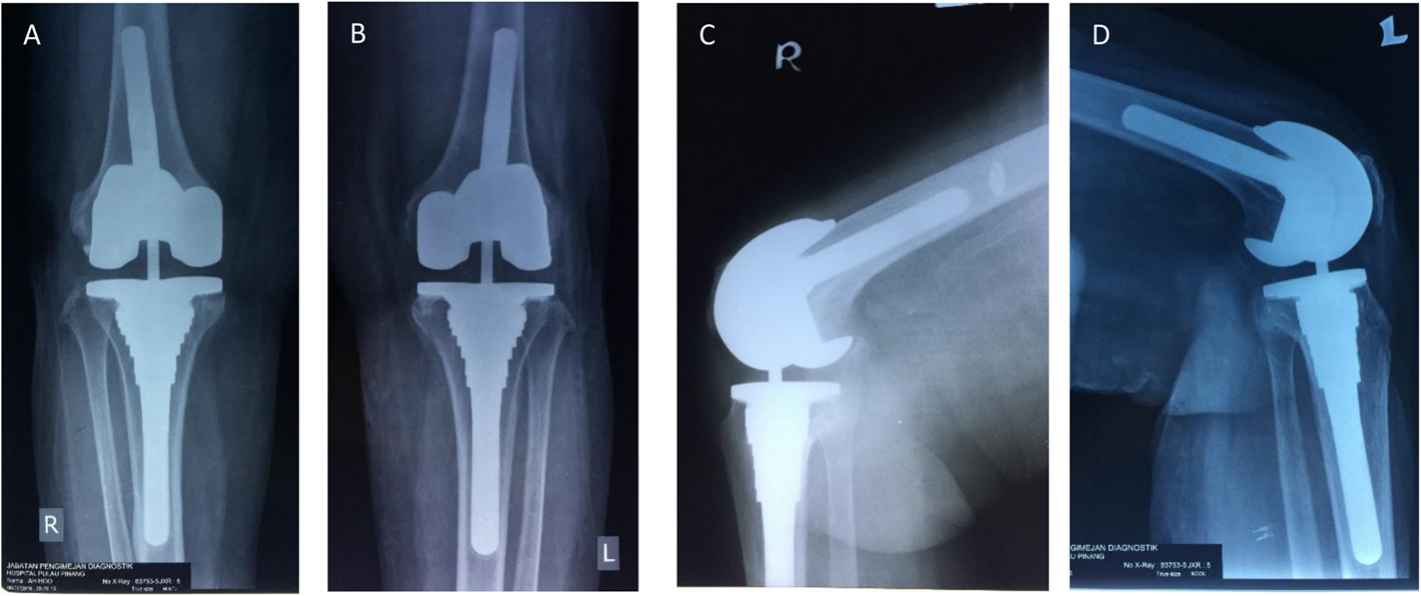
Post-op radiographs of one of our patients who had Type 2B defects bilaterally. **a** Anteroposterior (AP) Standing of Right knee. **b** Anteroposterior (AP) standing of left knee. **c** Lateral view of right knee. **d**. Lateral view of left knee

**Fig. 3:**
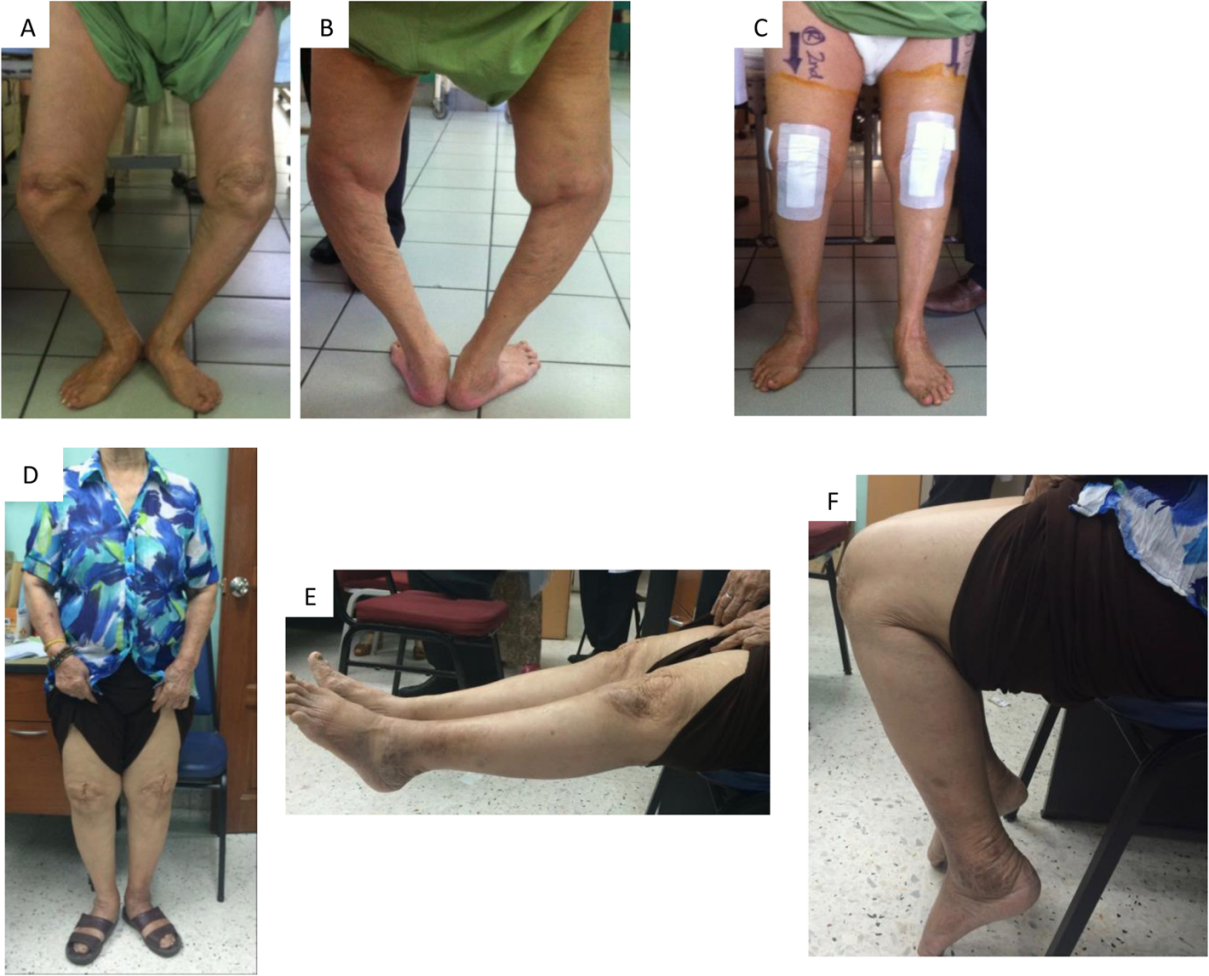
Pre-op and post-op clinical images of the same patient. **a** Anterior view of patient standing. **b** Posterior view of patient standing. **c** Anterior view of patient standing post-operatively. **d**–**f** Patient when we saw her at our follow-up clinic, demonstrating restoration of alignment and good range of motion

Patients were then reviewed and assessed at our clinic according to the aforementioned schedule. Figure 2 shows the postoperative radiographs for one of our patients and Fig. 3 depicts the clinical images of the patient pre-surgery and post-surgery. This study only includes the patients who have been followed up for a minimum of 2 years.

The updated (2011) KSS was used in conjunction with radiological assessments at each follow up. The KSS system was used as it not only takes into account the objective elements of alignment, stability and range of motion, which are determined by the surgeon, but also the subjective aspect of surgery whereby equal emphasis is placed on patient-reported outcomes as well. It is easily applicable across all types of knee replacement surgery and is a validated tool that has been widely used. Data were analysed using SPSS software version 15 (SPSS Inc., Chicago, IL, USA), using the paired sample t test for parametric data and Wilcoxon signed rank test for non-parametric data.

## Surgical technique

A midline skin incision was made over the affected knee and a medial parapatellar approach was employed in all patients. The femur and tibia were then prepared and bone cuts were made using the measured resection technique with the assistance of the cutting-jigs. The tibia was resected using an extramedullary jig by 9 mm based off the normal side, which is typically the lateral tibial plateau, as the patients in our series were mostly in severe varus. The extension gap is achieved by 9-mm distal femoral cut, and the flexion gap is achieved by 9-mm posterior femoral cuts, using intramedullary femoral jigs with a predetermined valgus angle for the distal cut and a posterior referencing sizer for the posterior condyle resections. Bone stock was then assessed to confirm whether a metaphyseal sleeve is needed. A starter reamer was used to open up the medullary canal before sequentially reaming to size for a snug diaphyseal fit of the tibial stem. This was then followed by sequential broaching of the metaphyseal bone until axial and torsional stability was achieved. Trial components were inserted and alignment, flexion-extension gap balance, mediolateral stability, range of motion and patellar tracking were assessed as standard. The mediolateral stability assessment was pivotal in deciding whether a normal posterior-stabilised (PS) construct would suffice or whether there was a need for a semi-constrained implant (TC3). Upon deciding to proceed with the TC3 implant, the femoral stem was further prepared by creating a larger notch cut. After bone resection, soft tissue release was carried out to achieve medio-lateral balance and to address the flexion-extension gap. As we are dealing specifically with complex primary knee osteoarthritis, the majority of our patients have a varus deformity with bone loss severely affecting the anteromedial and posteromedial portions of the tibial plateau. By referencing the joint line to the native lateral tibial plateau using the measured resection technique, whereby the amount of bone resected is measured and replaced by the implant, the native joint line is not altered. Final components are then inserted using the press-fit sleeves and medullary stems with cementation of the condylar portions of the femoral prosthesis and the tibial tray. Robert-Jones bandaging was applied for all patients and removed the following morning. No drains were inserted. Weight bearing with walking frames (4-legged walker) was commenced at day 1 post-surgery.

## Results

A total of 17 patients underwent surgery, 6 of them having bilateral knee replacement, bringing the total number of knees studied in our series to 23. Of the 23 knees studies, 13 were on the right side and 10 were on the left. Out of the 17 patients, 13 were female and 4 were male.

**Fig. 4:**
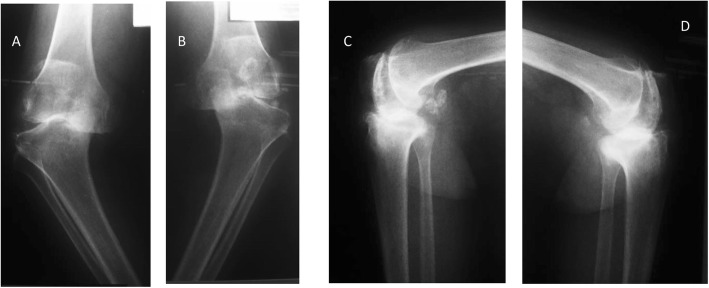
Pre-op radiographs of one of our patients who had Type 2B defects bilaterally. **a** Anteroposterior (AP) Standing of Right knee. **b** AP Standing of Left knee. **c** Lateral view of right knee. **d** Lateral view of left knee

Preoperative radiographs were assessed and graded using the AORI classification as outlined previously. Twelve knees were classified as type 2A and 11 knees were classified as type 2B. Figure 4 shows an example of one of our patients who had bilateral type 2B defects.

Mean KSS improved from 53.83 (S.D. = 28.88) preoperatively to 193.48 (S.D. = 19.39) postoperatively (*p* < 0.05) with functional scores improving from 17.78 (S.D. = 15.16) pre-surgery to 58.30 (S.D. = 9.58) post-surgery (*p* < 0.05) and these were statistically significant. Individual components are summarized in detail in Table 1. There was no significant difference when comparing post-surgical improvements in the mean KSS in the press-fit condylar (PFC) and TC3 groups (*p* = 0.78), with individual components shown in Table 2.

**Table 1:** Breakdown of results from the Knee Society Score Questionnaire

Category	Preop	Postop	***P*** value
	Mean (S.D.)	Mean (S.D.)	
Objective knee indicators	5.91 (15.46)	60.74 (17.72)	0.00003*^b^
Symptoms	5.70 (6.47)	23.78 (1.93)	0.00004*^b^
Patient satisfaction	14.00 (6.77)	37.04 (2.82)	0.00000000000002*^a^
Patient expectations	10.43 (1.70)	13.61 (1.31)	0.000009* ^a^
Functional score	17.78 (15.16)	58.30 (9.58)	0.00000000001* ^a^
Total	53.83 (28.88)	193.48 (19.39)	0.0000000000000007* ^a^

*S.D.* standard deviation, *Preop* Preoperative, *Postop* Postoperative

*Level of significance set at 0.05

^a^ Paired sample *t* test

^b^ Wilcoxon signed rank test

**Table 2:** Comparison of KSS Scores between PFC and TC3

Category	Preop	Postop	***P*** value
	Mean (S.D.)	Mean (S.D.)	
**Preop**			
Objective knee indicators	6.33 (18.30)	5.45 (12.52)	0.88^b^
Symptoms	4.33 (5.03)	7.18 (7.72)	0.30^a^
Patient satisfaction	16.00 (7.08)	11.82 (5.96)	0.14^a^
Patient expectations	10.50 (2.24)	10.36 (0.92)	0.85 ^a^
Functional score	16.33 (18.73)	19.36 (10.70)	0.64 ^a^
Total	53.50 (34.49)	54.18 (22.93)	0.96 ^a^
**Postop**			
Objective knee indicators	59.92 (19.50)	61.64 (16.45)	0.82^a^
Symptoms	23.42 (2.31)	24.18 (1.40)	0.35^a^
Patient satisfaction	37.67 (3.28)	36.36 (2.16)	0.28^a^
Patient expectations	13.67 (1.56)	13.55 (1.04)	0.83 ^a^
Functional score	57.58 (11.99)	58.91 (6.44)	0.75 ^a^
Total	192.25 (20.98)	194.64 (18.22)	0.78 ^a^

*S.D.* standard deviation, *Preop* Preoperative, *Postop* Postoperative

*Level of significance set at 0.05

^a^ Paired sample *t* test

^b^ Wilcoxon signed rank test

Knees were radiologically assessed by reviewing AP (standing) and lateral projection radiograph of the knee to assess alignment correction and presence of osseointegration or osteolysis. Mean alignment improved from a varus of 12.59° (S.D. = 15.41°) preoperatively to a valgus of 2.95° (S.D. = 3.48°) postoperatively (*p* < 0.05) and was statistically significant (Table 3). Osseointegration was defined as an absence of radiolucency lines between the host bone and implant (Mozella et al. [8]) and an increase in osteosclerosis at the bone-implant interface; all knees in our series demonstrated progressively increased osseointegration, especially between the radiographs taken at 6 weeks and 3 months post-surgery. No significant interval changes were noted on the radiographs acquired subsequently.

**Table 3:** Alignment and range of motion

Category	Preop	Postop	***P*** value
	Mean (S.D.)	Mean (S.D.)	
Alignment (*N* = 22)	12.59 (15.41)	−2.95 (3.48)	0.0003*^b^
ROM (*N* = 23)	87.00 (29.10)	105.87 (12.49)	0.003* ^a^

*S.D.* standard deviation, *Preop* Preoperative, *Postop* Postoperative, *ROM* range of motion

*Level of significance set at 0.05

^a^ Paired sample *t* test

^b^ Wilcoxon signed rank test

Range of motion also improved from 87.00° (S.D. = 29.10°) to 105.87° (S.D. = 12.49°), (*p* < 0.05) with 19 out of the 23 knees achieving full extension (82.6%). This improvement in range of motion was statistically significant and is depicted in Table 3. There were no infected implants or any with septic/aseptic loosening. No patient complained of either anterior knee pain or end of stem pain and there were no patients who sustained a periprosthetic fracture. To date, none of our patients have required revision surgery.

## Discussion

TKR is an established treatment option in knee osteoarthritis, with excellent results and reproducibility (Meding et al. [3]). Issues begin to arise for the general orthopaedic surgeon when the affected knee has significant bone loss, extreme varus/valgus deformity, contractures or ligamentous instability (Baldini et al. [4]), rather than straightforward simple primary osteoarthritis. The issue we are attempting to address is the significant bone loss. There are currently no reports available on a single method that has proven to be superior to others, and decisions on treatment modality are still dependent largely on the expertise and implants at hand. They include using bone cement either on its own or in conjunction with screws (Glynn and Austin [9]), bone grafting (Goldstein et al. [10], Lonner et al. [11]), metal augments (long stems, wedge augments, tantalum cones, metaphyseal sleeves) (Glynn and Austin [9], Mozella et al. [8], Fedorka et al. [12], Dalury and Barrett [13], Bugler et al. [14], Huang et al. [15], Vasso et al. [16], Barnett et al. [17], Brown et al. [18], Gross and Liu [19]), structural allografts (Glynn and Austin [9], Engh and Ammeen [20], Backstein et al. [21], Kuchinad et al. [22]), hinged prostheses (Glynn and Austin [9], Sanguineti et al. [23]) and custom-made implants (Glynn and Austin [9]). The metaphyseal sleeve is one such option in revision TKR; however, to the author’s knowledge it has not been described as a treatment option for cases of complex primary knee osteoarthritis.

Cementation alone is usually reserved for small contained defects with a depth of less than 5 mm and if the defect is uncontained, a screw and cement construct would be necessary [9]. Benefits of using cement, with or without screws are that they are inexpensive, readily available and are easily contoured to fill the defects; however, these are for small and shallow defects and will not be suitable for knees with massive bone loss, which we have attempted to address in this study [9].

Metaphyseal sleeves are attractive as they overcome the issue of inadequate bone stock in the epiphyseal region by directly forming contact with the metaphysis. This confers enhanced load distribution and sharing between bone and implant. This enhanced load channelling to metaphyseal bone makes this preferable over utilising a stem and wedge construct. The ability to independently rotate the metaphyseal sleeve to the best bone stock available also has the added advantage of permitting the desired alignment correction to be achieved rather easily. The coupling of the metaphyseal sleeve with an extra intra-medullary stem further augments the stability of the implant and whilst there have been cases where end-of-stem pain has been reported in other studies, none of our patients have reported as such (Glynn and Austin [9], Fedorka et al. [12], Dalury and Barrett [13], Bugler et al. [14], Huang et al. [15], Vasso et al. [16], Barnett et al. [17]). A wedge augment will also confer load transfer from implant to bone and permit immediate weight bearing as per the metaphyseal sleeve, but its disadvantages include the limitations in size and shape and its unsuitability for use when the bone defects are massive.

Allografts and autografts are treatment options in AORI types 2A/2B or 3. In comparison with the use of allografts, the metaphyseal sleeve does not confer any risk of disease transmission (Vasso et al. [16], Daines and Dennis [24]) and there would be a lower risk of infection, given that infection is well-documented as a complication of allografts, presumably due to the prolonged duration of surgery where meticulous preparation is required (Vasso et al. [16], Engh and Ammeen [20]). The availability of allografts is another issue to consider. The metaphyseal sleeves are more readily available as compared to allografts, albeit they are more costly (Vasso et al. [16], Daines and Dennis [24]). With regards to autograft, this is usually procured from the bone cuts made during preparation of the femur and tibia, and the inherent uncertainty about the bone quality and the amount of graft available has to be considered. The use of grafts mandates that osseointegration takes place before the patient is allowed to ambulate but immediate ambulation can be commenced if using the metaphyseal sleeves, thus circumventing the morbidities associated with prolonged immobilization, such as development of pressure sores, deep vein thrombosis and pneumonia. Autografts do confer the theoretical possibility of restoring bone stock and are economically cheaper than the metaphyseal sleeve; however, the risk of bone resorption has to be factored in as well.

The tantalum cone is an enticing option especially for centrally based defects; however, it is merely a bone-gap filler. The coupling mechanism between the metaphyseal sleeve and the diaphyseal stem as per our construct confers substantially enhanced load channelling as compared to the tantalum cones. Additionally, the hydroxyapatite-coated surfaces of our metaphyseal sleeves can attain desirable osseointegration, which in our opinion is comparable with that of the tantalum cones, as has been highlighted as one of the notable benefits when using the tantalum cones (Mozella et al. [8], Fedorka et al. [12], Brown et al. [18]). The broaching technique used to prepare the metaphysis for the metaphyseal sleeve also ensures that there is good bone-implant contact and that there is no need for additional bone grafting, which is required to fill up gaps between the tantalum cones and adjacent bone as reported in two previously published series (Long and Scuderi [25], Meneghini et al. [26]).

Implant longevity will always be an issue to consider and the lesser the constraint required to achieve stability, the better the ultimate longevity of the implant. Whilst being able to accord a significant degree of constraint when using the TC3 implant, it would be less than that of a hinged prosthesis. This would inevitably result in less wear, thus theoretically prolonging implant survival.

The main drawback of using the metaphyseal sleeve would be, in our opinion, the process of removal of the entire implant should such a need arise due to complications (Daines and Dennis [24]). However, there is already a removal system in place and explantation of the entire construct would be rather straightforward with the use of this system, and the knee would then be treated as per any other revision TKR in determining further courses of action.

The other drawback would be the inability to use an off-set stem to centralize the tibial tray in attempts to increase load surface coverage but as aforementioned, the fact that the load is channelled directly to the metaphyseal bone renders this drawback a non-issue. The appearance of a non-centrally placed tibial tray should not be cause for alarm when reviewing postoperative radiographs.

The risk of metallosis as a result of the broaching technique in preparing the metaphysis for the sleeve is a known risk factor; however, Jones et al. [27] have published a 2-year study on 16 knees that were treated using the S-ROM mobile-bearing hinged prosthesis, which also required sequential broaching. In their study, there were no reported cases of metallosis, which is in keeping with the results of our study whereby there have been no cases of clinically significant metallosis to date either.

Limitations of this study include the fact that there is no control group and as such we are not able to ascertain whether the metaphyseal sleeve is directly superior over another treatment option, and as this was a retrospective study, randomization was not possible. Although the results of this study are very promising, the follow-up period of 2 years would be deemed as short-term and a longer duration of follow up would be necessary to ascertain the long-term outcomes of the metaphyseal sleeve.

The results of our study in terms of clinical and radiological improvement are comparable with those in the published studies on revision TKR (Fedorka et al. [12], Bugler et al. [14], Huang et al. [15], Barnett et al. [17], Alexander et al. [28], Martin-Hernandez et al. [29], Graichen et al. [30]) and this study demonstrates that excellent results are attainable when the sleeve is employed in the treatment of complex primary TKR. The improvement in symptoms, range of motion and degree of functional activities that could be performed by our patients enabled a return to a quality of life that had long been hampered by severe osteoarthritis.

## Conclusion

The metaphyseal sleeve is an excellent treatment option for complex primary knee osteoarthritis, with good results objectively, functionally and radiologically, and would be a great choice to include in the armamentarium of all orthopaedic surgeons.

## Data Availability

The datasets collected and analysed during the current study are available from the corresponding author on reasonable request.

## Abbreviations

AORI: Anderson Orthopaedic Research Institute
KSS: Knee Society Score
PFC: Press-fit condylar
ROM: Range of motion
S.D.: Standard deviation
TC3: Semi-constrained implant
TKR: Total knee replacement(s)
